# HB-EGF–EGFR Signaling in Bone Marrow Endothelial Cells Mediates Angiogenesis Associated with Multiple Myeloma

**DOI:** 10.3390/cancers12010173

**Published:** 2020-01-10

**Authors:** Luigia Rao, Donato Giannico, Patrizia Leone, Antonio Giovanni Solimando, Eugenio Maiorano, Concetta Caporusso, Loren Duda, Roberto Tamma, Rosanna Mallamaci, Nicola Susca, Alessio Buonavoglia, Matteo Claudio Da Vià, Domenico Ribatti, Vallì De Re, Angelo Vacca, Vito Racanelli

**Affiliations:** 1 Department of Biomedical Sciences and Human Oncology, Guido Baccelli Unit of Internal Medicine, University of Bari Medical School, 70124 Bari, Italy; luigiarao@gmail.com (L.R.); dani.ezio@hotmail.it (D.G.); patrizia.leone@uniba.it (P.L.); antoniogiovannisolimando@gmail.com (A.G.S.); susnic@libero.it (N.S.); alessio.buonavoglia85@gmail.com (A.B.); angelo.vacca@uniba.it (A.V.); 2 Department of Emergency and Organ Transplantations, Section of Pathological Anatomy, University of Bari Medical School, 70124 Bari, Italy; eugenio.maiorano@uniba.it; 3 Department of Interdisciplinary Medicine, University of Bari Medical School, 70124 Bari, Italy; concetta.caporusso@policlinico.ba.it (C.C.); lorenduda@live.it (L.D.); 4 Department of Basic Medical Sciences, Neurosciences, and Sensory Organs, Section of Human Anatomy and Histology, University of Bari Medical School, 70124 Bari, Italy; roberto.tamma@uniba.it (R.T.); domenico.ribatti@uniba.it (D.R.); 5 Department of Biosciences, Biotechnology and Biopharmaceutics, University of Bari, 70124 Bari, Italy; rosanna.mallamaci@uniba.it; 6 Department of Internal Medicine II, University Hospital Würzburg, 97080 Würzburg, Germany; davia_m@ukw.de; 7 Bio-Proteomics Facility, Department of Translational Research, Centro di Riferimento Oncologico di Aviano (CRO) IRCCS, 33081 Aviano (PN), Italy; vdere@cro.it; 8 Vito Racanelli MD, Department of Biomedical Sciences and Human Oncology, University of Bari Medical School, Policlinico, 11 Piazza G. Cesare, 70124 Bari, Italy

**Keywords:** multiple myeloma, HB-EGF, EGFR, bone marrow angiogenesis, endothelial cells

## Abstract

Epidermal growth factor receptor (EGFR) and its ligand heparin-binding EGF-like growth factor (HB-EGF) sustain endothelial cell proliferation and angiogenesis in solid tumors, but little is known about the role of HB-EGF–EGFR signaling in bone marrow angiogenesis and multiple myeloma (MM) progression. We found that bone marrow endothelial cells from patients with MM express high levels of EGFR and HB-EGF, compared with cells from patients with monoclonal gammopathy of undetermined significance, and that overexpressed HB-EGF stimulates EGFR expression in an autocrine loop. We also found that levels of EGFR and HB-EGF parallel MM plasma cell number, and that HB-EGF is a potent inducer of angiogenesis in vitro and in vivo. Moreover, blockade of HB-EGF–EGFR signaling, by an anti-HB-EGF neutralizing antibody or the EGFR inhibitor erlotinib, limited the angiogenic potential of bone marrow endothelial cells and hampered tumor growth in an MM xenograft mouse model. These results identify HB-EGF–EGFR signaling as a potential target of anti-angiogenic therapy, and encourage the clinical investigation of EGFR inhibitors in combination with conventional cytotoxic drugs as a new therapeutic strategy for MM.

## 1. Introduction

Epidermal growth factor receptor (EGFR, also known as ErbB-1 and HER1) belongs to the ErbB family of receptor tyrosine kinases, together with ErbB-2 (neu, HER2), ErbB-3 (HER3) and ErbB-4 (HER4) [1]. In normal tissues, EGFR modulates cell growth and differentiation, but in tumors its aberrant expression or activity can contribute to an aggressive phenotype and poor prognosis [2,3,4]. EGFR overexpression or overactivation can result from mutations locking the receptor in a state of continual signaling or from abnormally high levels of EGFR ligands released from tumor cells or non-tumor cells of the tumor microenvironment [5]. These ligands include epidermal growth factor (EGF), transforming growth factor (TGF)-α, heparin-binding EGF-like growth factor (HB-EGF), betacellulin, amphiregulin (AREG), and epiregulin (EREG) [6]. The interaction of EGFR with its ligands leads to receptor homodimerization or heterodimerization with another member of the ErbB family. Dimerization causes EGFR autophosphorylation at specific tyrosine residues in its intracellular domain, triggering downstream signaling pathways that are often overactivated in malignant cells during tumor progression [7].

In cancer therapy, EGFR signaling can be pharmacologically inhibited by directly blocking the extracellular ligand-binding site (as does the monoclonal antibody cetuximab) or the intracellular kinase domain (as do the small molecules gefitinib and erlotinib) [8]. Moreover, EGFR activation can be prevented by drugs that neutralize EGFR ligands in the tumor microenvironment, as has been demonstrated for an antibody against HB-EGF in ovarian cancer [9]. HB-EGF is initially synthesized as a membrane-anchored 23 kDa protein that is later processed by several matrix metalloproteinases (MMPs) and ADAM metallopeptidases into 12-to-22 kDa soluble forms that specifically bind EGFR (HER1) and HER4 in both autocrine and paracrine fashions [10]. HB-EGF–EGFR signaling has been implicated in carcinogenesis and progression of solid tumors, such as pancreas and lung cancer [11,12]. 

Little is known about HB-EGF–EGFR signaling in hematological cancers, such as multiple myeloma (MM), a lethal malignancy of bone marrow plasma cells that is often anticipated by a preneoplastic phase termed monoclonal gammopathy of undetermined significance (MGUS) [13]. One study reported that HB-EGF treatment of EGFR-expressing MM plasma cell lines improved the pro-survival effects of interleukin-6 [14]. In a successive study by the same group, an inhibitor of ErbB family receptor tyrosine kinases promoted primary MM plasma cells to enter apoptosis and strengthened the apoptotic effects of dexamethasone or of an interleukin-6 neutralizing antibody [15]. A third study showed that bone marrow stromal cells from MM patients produced HB-EGF and expressed high mRNA levels of all the ErbB receptor tyrosine kinases except for HER4, while primary MM plasma cells did not express the *HBEGF* gene but did express variable levels of EGFR (HER1) mRNA and HER4 mRNA [16]. It is; therefore, likely that HB-EGF–EGFR signaling is a major mediator of the cross-talk between MM plasma cells and cells of the bone marrow stroma, including endothelial cells.

HB-EGF is known to sustain endothelial cell proliferation and angiogenesis in ovarian and bladder cancer [17,18]. This effect; however, has not yet been investigated in MM where bone marrow endothelial cells are known to be powerful promoters of bone marrow angiogenesis and MM progression [19,20]. Indeed, the extent of bone marrow angiogenesis at the time of MM diagnosis has become a predictive factor for disease progression [21], and angiogenesis-targeting therapies have emerged as important tools for improving MM treatment [22,23]. The present study was; therefore, conducted to test the hypotheses that HB-EGF–EGFR signaling is involved in bone marrow angiogenesis and that its blockade prevents MM progression. 

## 2. Results

To test the hypothesis that HB-EGF–EGFR signaling drives bone marrow angiogenesis and promotes MM progression, we studied the expression and activity of these proteins in bone marrow cells and tissues from MM and MGUS patients.

### 2.1. EGFR Expression

**Figure 1:**
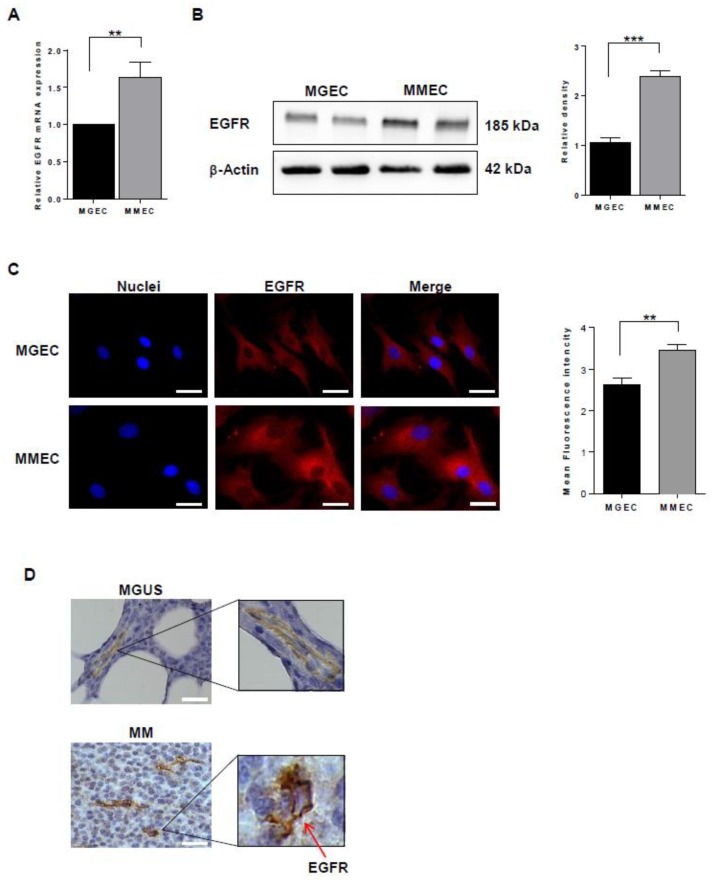
EGFR expression is higher in bone marrow endothelial cells from MM than MGUS patients. (**A**) Relative mRNA levels of epidermal growth factor receptor (EGFR) in endothelial cells from MGUS and MM patients (MGEC and MMEC, respectively), determined by real time-PCR. Samples from six MGUS and six MM patients were tested in triplicate. Data are expressed as mean and SD. (**B**) Western blot of EGFR (using a rabbit anti-human antibody) and β-actin in whole cell lysates of MGEC and MMEC (**left**) and results of densitometric analysis, with EGFR values normalized first to β-actin and then to MGEC values (**right**). Samples from eight MGUS and eight MM patients were tested in triplicate. Values are expressed as mean and SD. (**C**) Immunofluorescence staining of EGFR (using a rabbit anti-human antibody; red) on cultured MGEC and MMEC (**left**) and quantification analysis (**right**). DAPI (blue) was used to stain nuclei. Control experiments without the primary antibody (omitted) showed no background staining. Representative photomicrographs of four independent experiments are shown. Original magnification 400×. Scale bar, 25 μm. The quantification of the immunofluorescence was performed by ImageJ software. (**D**) Immunohistochemical detection of EGFR (pink) on CD31-positive cells (brown) in bone marrow vessel walls from MGUS and MM patients. The images were analyzed by two independent pathologists in a blind fashion. Representative photomicrographs of four independent experiments are shown. Original magnification 400×. Scale bar, 25 μm. ** *p* < 0.01 and *** *p* < 0.001, Mann–Whitney U test.

First, we examined the expression of EGFR in primary endothelial cells from MGUS and MM patients (MGEC and MMEC, respectively). EGFR mRNA levels were significantly lower in MGEC than MMEC (Figure 1A). Similarly, EGFR protein levels were lower in MGEC than MMEC, as shown by both Western blotting (Figure 1B) and immunofluorescence (Figure 1C). Working with bone marrow tissue ex vivo, we observed EGFR expression on vessel walls in sections from MM patients but not from MGUS patients (Figure 1D). These results indicate that EGFR is expressed by bone marrow endothelial cells, at low levels in MGUS patients and at higher levels in MM patients.

**Figure 2:**
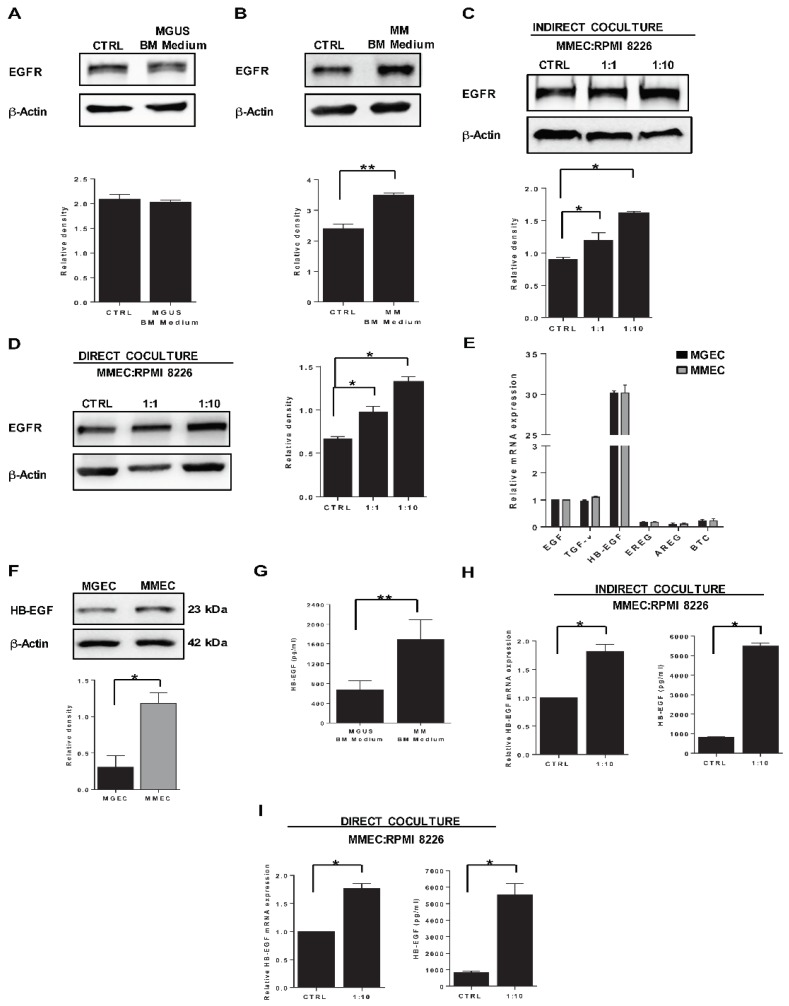
Bone marrow microenvironment influences EGFR and HB-EGF expression in MMEC. Western blots of EGFR and ≥β-actin in MMEC and results of densitometric analyses of EGFR normalized to β-actin. (**A**,**B**) MMEC were maintained for 24 h in normal culture medium (CTRL) or culture medium conditioned by bone marrow mononuclear cells (BMMC) from (**A**) MGUS patients (MGUS BM medium) or (**B**) MM patients (MM BM medium). (**C**,**D**) MMEC were grown for 24 h, alone (CTRL) or in coculture with RPMI 8226 cells at 1:1 and 1:10 cell ratios, separated (**C**) or not (**D**) by Transwell inserts. All charts report data from six patients. Each sample was tested in triplicate with similar results. (**E**) Basal relative mRNA levels of six EGFR ligands in MGEC and MMEC, determined by real-time PCR. Values were normalized to that of EGF. Samples from six MGUS and six MM patients were tested in triplicate. (**F**) Western blots of HB-EGF and β-actin in MGEC and MMEC, and results of densitometry analysis of HB-EGF, normalized to β-actin. Samples from six MGUS and six MM patients were tested in triplicate. (**G**) ELISA determination of HB-EGF in conditioned culture medium from BMMC of 12 MGUS and 12 MM patients. (**H**,**I**) Relative HB-EGF mRNA levels (**left**) and ELISA determinations of soluble HB-EGF in conditioned medium (**right**) of MMEC cultured for 24 h, alone (CTRL) or in coculture with RPMI 8226 cells, indirectly (**H**) or directly (**I**). Samples from six patients were tested in triplicate. Values are expressed as mean and SD, * *p* < 0.05 and ** *p* < 0.01, Mann–Whitney U test.

To investigate if EGFR expression is influenced by the bone marrow microenvironment, we treated MMEC with conditioned culture media from bone marrow mononuclear cells (BMMC) of MGUS and MM patients. EGFR protein levels were unaffected by medium conditioned by MGUS BMMC (Figure 2A), while they increased during treatment with medium conditioned by MM BMMC (Figure 2B). To determine if the observed increase in EGFR level was driven by tumor plasma cells and related to tumor progression, we set up a coculture experimental system mimicking interactions between endothelial and plasma cells during tumor growth. Specifically, we cocultured MMECs with increasing numbers of Roswell park memorial institute (RPMI) 8226 cells (a tumor plasma cell line), together and separated by a Transwell membrane (direct and indirect cocultures, respectively). Western blotting showed that EGFR protein levels were upregulated in both coculture conditions (with and without the Transwell membrane) in a manner proportional to the number of RPMI 8266 cells present (Figure 2C,D). These results indicate that, in MM but not MGUS, EGFR expression by bone marrow endothelial cells is influenced by soluble factors released by plasma cells during tumor progression.

### 2.2. HB-EGF Expression

To investigate whether MGEC and MMEC express HB-EGF or other EGFR ligands (EGF, TGF-α, epiregulin, amphiregulin, and betacellulin), we determined relative mRNA levels of the corresponding genes. Levels of *HBEGF* mRNA were high compared to those of the five other EGFR ligands, in both MGEC and MMEC, with no significant difference between cell types (Figure 2E). Focusing on HB-EGF, we found higher steady-state levels of the full-length (uncleaved) protein in MMEC than in MGEC (Figure 2F). These results suggest that HB-EGF is an important autocrine EGFR ligand in these cells.

Next, to assess whether the expression of EGFR in cells exposed to conditioned medium was associated with different levels of HB-EGF in the latter, we determined the concentration of soluble HB-EGF in MGUS BM medium and MM BM medium and found significantly higher levels in the medium from BMMC from MM patients (Figure 2G). To determine if HB-EGF expression in MMEC was modulated also in our coculture experimental settings, we measured its mRNA and protein levels in cocultures with RPMI 8226 cells. These experiments showed significantly higher levels of HB-EGF mRNA in MMEC and of soluble HB-EGF protein in culture medium, in both indirect cocultures separated by a Transwell membrane (Figure 2H) and direct cocultures (Figure 2I).

Because soluble HB-EGF is cleaved from the plasma membrane by several proteases, we assessed whether the expression of these enzymes in MMEC was promoted by tumor plasma cells. In particular, we measured the mRNA levels of MMP3, MMP7, ADAM10, ADAM12, and ADAM17 in MMEC grown in monoculture and cocultured with RPMI 8226 cells, together or separated by a Transwell membrane. Differences in relative mRNA levels between the monoculture and the coculture were observed only for MMP7, which had higher levels in coculture than in monoculture (Figure S1). The increase in MMP7 levels relative to control monocultures was greater in direct than indirect cocultures. These results suggest that MM plasma cells are responsible for EGFR upregulation on endothelial cells by promoting MMP7-mediated cleavage of HB-EGF from the surface of the same cells.

### 2.3. Role of HB-EGF in MM-Associated Angiogenesis In Vitro

**Figure 3:**
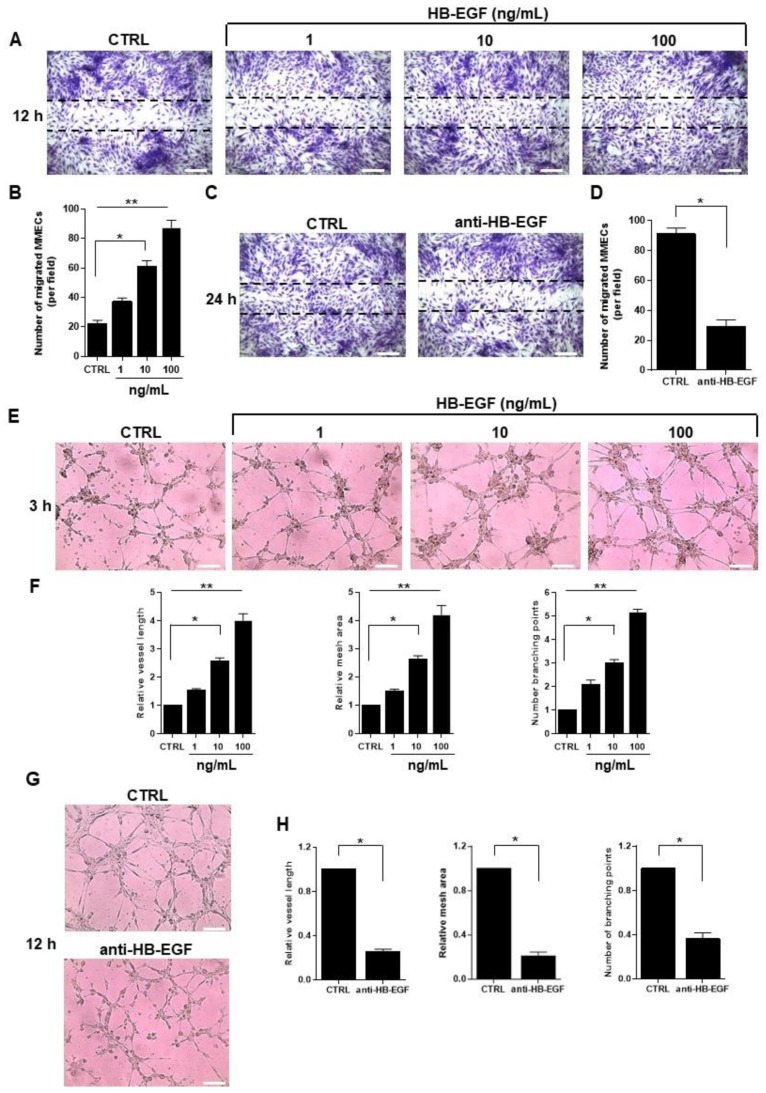
HB-EGF stimulates in vitro MMEC migration and angiogenesis in a dose-dependent manner. (**A**–**D**) Wound-healing assay. (**A**) Photomicrographs of MMEC in serum-free medium with increasing concentrations of soluble recombinant HB-EGF, 12 h after confluent monolayers were wounded by scraping with MMEC in serum-free medium with increasing concentrations of soluble recombinant HB-EGF. (**B**) Counts of migrating cells in each wound of (**A**), for six independent experiments. (**C**) MMEC were wounded and treated with serum-free medium alone or with 0.5 µg/mL neutralizing anti-HB-EGF antibody. (**D**) Counts of migrating cells in each wound of (**C**), for six independent experiments. (**E**–**H**) Matrigel angiogenesis assay. (**E**) Photomicrographs of MMEC, 3 h after seeding on Matrigel in serum-free medium with increasing concentrations of HB-EGF. Images are representative of experiments using cells from six patients. (**F**) Quantification of angiogenic behavior in (**E**) by topological analysis. (**G**) MMEC were seeded on Matrigel in the absence (CTRL) or presence of 0.5 µg/mL anti-HB-EGF antibody and photographed 12 h later. Images are representative of experiments on samples from six patients. (**H**) Quantification of angiogenic behavior in (G) by topological analysis. Original magnification, 200×. Scale bar, 50 μm. Data are mean and SD of six independent experiments. * *p* < 0.05 and ** *p* < 0.01, Mann–Whitney U test.

Based on the previous observations, we hypothesized that the increase in HB-EGF expression in conditions mimicking endothelial-tumor cell interactions is responsible for boosting angiogenesis. To begin to test this hypothesis, we treated MMEC with supernatant of RPMI 8226 cocultures (Figure S2) and increasing concentrations of recombinant HB-EGF, and assessed angiogenic functions in vitro (Figure 3). 

In a wound-healing assay, 12 h after monolayers were wounded by scraping, MMEC were seen to have migrated into the wound at numbers that increased with the concentration of HB-EGF in the medium (Figure 3A). This observation was confirmed by the counts of MMEC that had migrated into the wound (Figure 3B). To determine if HB-EGF directly affects MMEC’s spontaneous migration, the wound healing assay was repeated without added HB-EGF, and in the absence or presence of a neutralizing anti-HB-EGF antibody to block endogenously produced HB-EGF. After 24 h, MMEC migration was found to be significantly impaired in samples treated with the antibody, compared to controls (Figure 3C,D).

Similar results were obtained in an in vitro angiogenesis assay where MMEC were plated on Matrigel-coated plates in serum-free medium with increasing concentrations of HB-EGF (Figure 3E). After 3 h, the control sample appeared at the initial stage of organization, with small clumps of cells distributed on the Matrigel, whereas in HB-EGF-treated samples a structured capillary network was observed, with greater complexity at higher concentrations. The complexity of the structure was directly proportional to the HB-EGF concentration as shown by significant increases in the topological parameters vessel length, mesh area, and number of branching points (Figure 3F). In the presence of a neutralizing anti-HB-EGF antibody, capillary network formation was impaired (Figure 3G) and, compared to control samples, values of all three topological parameters were significantly lower (Figure 3H). Overall, these data suggest that HB-EGF is a potent inducer of angiogenesis in vitro and that blockade of autocrine HB-EGF halts the angiogenic capability of MMEC.

To examine whether and how HB-EGF modulates the angiogenesis-related protein profile of MMEC, serum-free culture media conditioned by MMEC, in the presence or absence of exogenous soluble recombinant HB-EGF, were analyzed by sandwich immunoassay (Figure S3). This analysis showed that the addition of HB-EGF to the culture medium upregulated several molecules with pro-angiogenic activity, thus explaining the MMEC behavior observed in vitro.

### 2.4. Role of HB-EGF in MM-Associated Angiogenesis In Vivo

**Figure 4:**
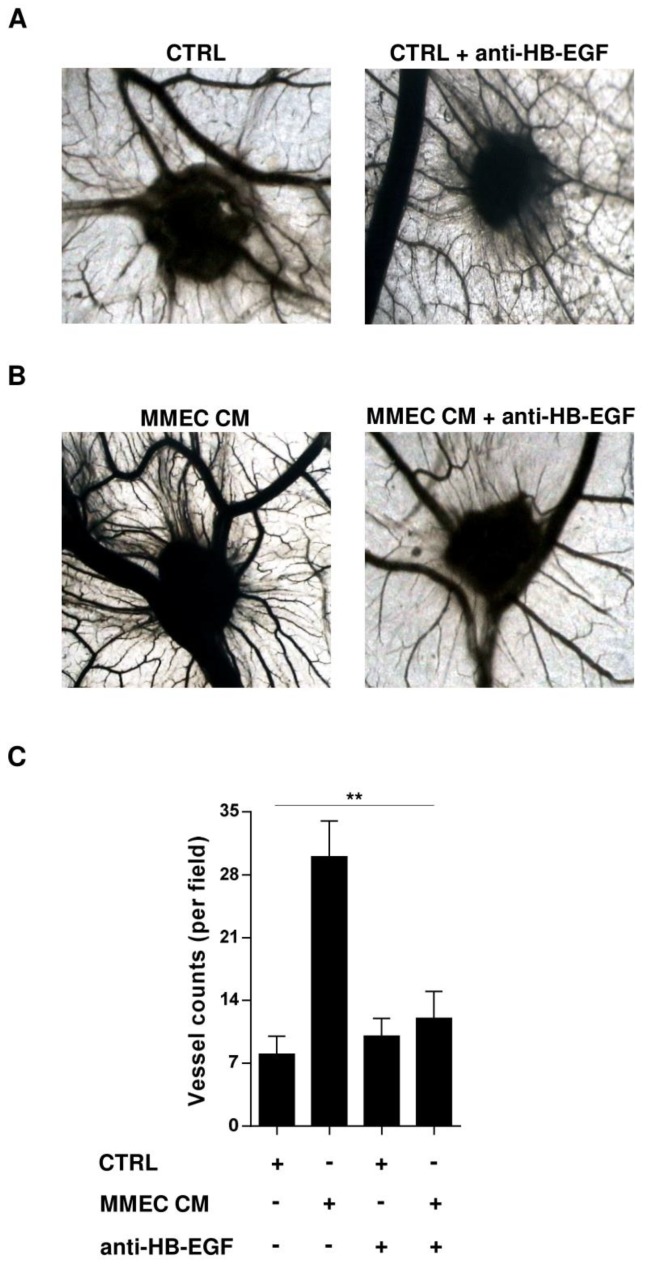
HB-EGF stimulates MM-associated angiogenesis in vivo. (**A**,**B**) Representative photographs taken in ovo of the chorioallantoic membrane assay on day 12. (**A**) Gelatin sponges soaked with serum-free medium, alone (**left**, CTRL) or with 0.5 µg/mL anti-HB-EGF antibody (**right**). (**B**) Gelatin sponges soaked with MMEC-conditioned serum-free medium (left, MMEC CM) or with MMEC-conditioned serum-free medium containing 0.5 µg/mL HB-EGF antibody (right). Original magnification 50×. (**C**) Quantification of newly formed vessels. Results are mean and SD of three technical replicates. ** *p* < 0.01, Mann–Whitney U test.

Based on the in vitro observations, we used the chorioallantoic membrane assay to investigate whether HB-EGF influences MM-associated angiogenesis in vivo. When egg chorioallantoic membranes were implanted with gelatin sponges loaded with serum-free culture medium, either fresh or conditioned by MMEC, many more new blood vessels formed in the presence of the conditioned medium (Figure 4A,B). When the experiment was repeated with addition of a neutralizing anti-HB-EGF antibody, the effect of MMEC-conditioned medium on new vessel growth was lost, while there was no noticeable change in control samples (Figure 4A–C). These results suggest that the angiogenic effects of MMEC-conditioned medium are due to the presence of HB-EGF.

### 2.5. Impact of EGFR Blockade on MM-Associated Angiogenesis

Because HB-EGF is able to bind both EGFR and HER4, we measured HER4 mRNA levels in MGEC and MMEC and found higher levels in MGEC than in MMEC (Figure S4A). HER4 protein levels were similar in MGEC and MMEC (Figure S4B). Moreover, HER4 protein levels in MMEC were not influenced by co-culturing with RPMI 8226 cells (Figure S4C). These data suggest that HB-EGF’s effects on MMEC are mediated solely by EGFR.

**Figure 5:**
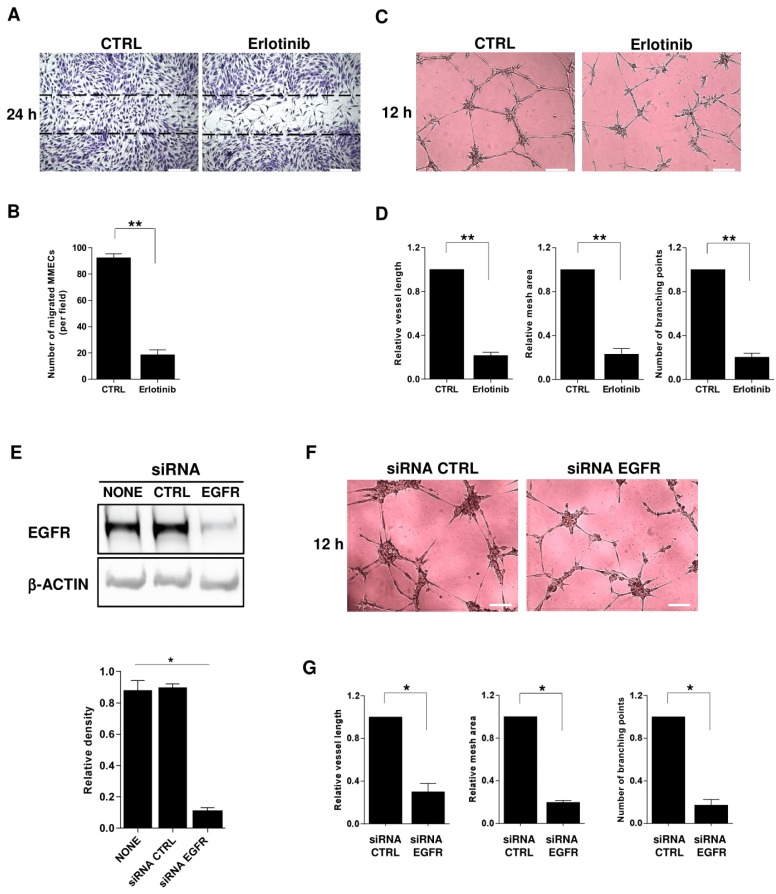
Erlotinib restrains the in vitro angiogenic potential of MMEC. (**A**,**B**) Wound-healing assay. (**A**) Photomicrographs of MMEC in serum-free medium without (CTRL) or with 10 µM erlotinib, 24 h after wounding. (**B**) Counts of migrating cells in each wound of (A). (**C**,**D**) Angiogenesis assay. (**C**) Photomicrographs of MMEC, 12 h after seeding on Matrigel without (CTRL) or with 10 µM erlotinib. (**D**) Quantification of angiogenic behavior in (**E**) by topological analysis. (**E**) Western blot of MMEC transfected with no nucleotides (CTRL), 50 nM control, non-targeting siRNA, or 50 nM EGFR siRNA for 48 h, and densitometric analysis of EGFR normalized to β-actin. (**F**) Photomicrographs of MMEC transfected with EGFR siRNA or control non-targeting siRNA seeded on Matrigel for 12 h. Images are representative of six experiments. (**G**) Quantification of angiogenic behavior in (**F**) by topological analysis. Data are mean and SD of six independent experiments. * *p* < 0.05 and ** *p* < 0.01, Mann–Whitney U test.

To further investigate the role of HB-EGF–EGFR signaling in MM, we repeated the in vitro functional assays with pharmacological blocking of EGFR by erlotinib, which specifically inhibits the receptor’s tyrosine kinase. In a wound-healing assay, erlotinib-treated MMEC migrated less than untreated MMEC (Figure 5A,B). In an in vitro angiogenesis assay, adding erlotinib to the medium prevented MMEC from forming a structured capillary network, with broken connections and isolated cells remaining (Figure 5C,D). 

To firmly establish the EGFR-dependent angiogenic effect, we used RNA interference to silence the receptor. EGFR siRNA efficiently knocked down EGFR protein expression in MMEC compared with untreated MMEC and MMEC treated with control non-targeting siRNA (Figure 5E). In the in vitro angiogenesis assay, EGFR siRNA-transfected MMEC did not form a structured capillary network (Figure 5F,G).

### 2.6. Anti-Angiogenic Effect of Erlotinib in MM Mouse Model

**Figure 6:**
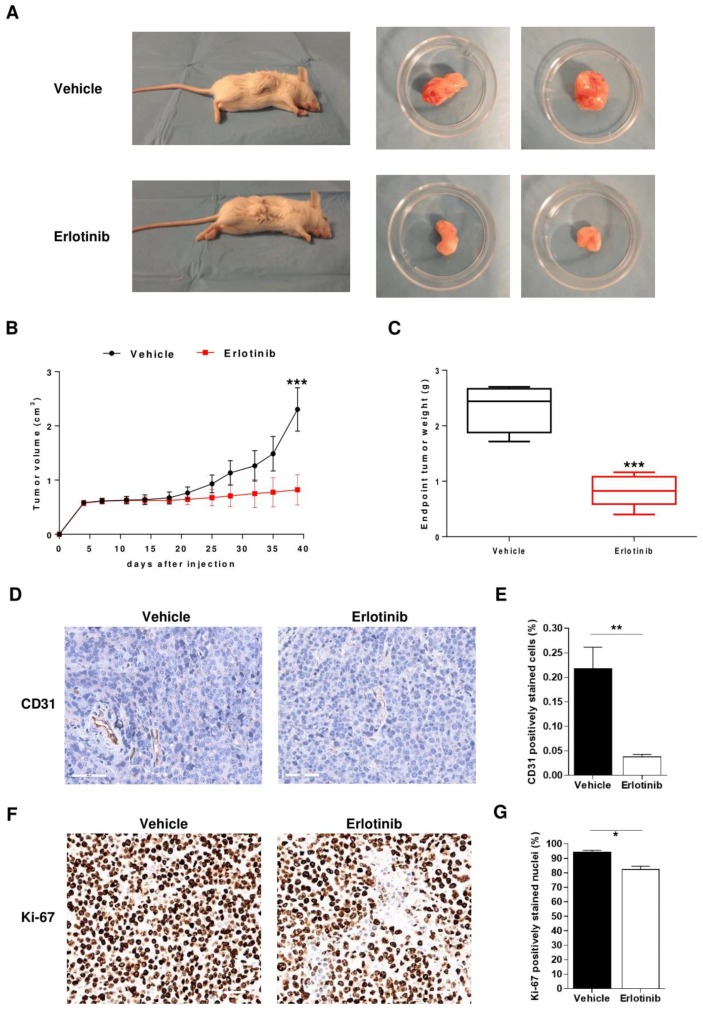
Erlotinib halts in vivo angiogenesis and suppresses tumor growth in an MM xenograft mice model. Non-obese diabetic mice with severe combined immunodeficiency (NOD/SCID) bearing RPMI 8226 xenografts were treated intraperitoneally with 50 mg/kg erlotinib (*n* = 10) or vehicle (*n* = 10). (**A**) Representative images of mice and excised tumors. (**B**) Change in tumor volume over time. (**C**) Estimated weights of excised tumors on day 40. Data are expressed as median, interquartile range (box), and range (whiskers). (**D**,**E**) Tumor sections stained for the microvessel density marker CD31 and quantification of positively stained murine endothelial cells. (**F**,**G**) Tumor sections stained for the proliferative marker Ki-67 and quantification of positively stained nuclei. Immunohistochemical images were analyzed by two independent pathologists in a blind fashion. Immunohistochemical quantifications are the average of five slides for each tumor and five fields per slide. * *p* < 0.05, ** *p* < 0.01, *** *p* < 0.001, Mann–Whitney U test.

To investigate whether EGFR inhibition affects in vivo angiogenesis and in turn MM development, we used a MM mouse model in which non-obese diabetic mice with severe combined immunodeficiency (NOD/SCID) were subcutaneously injected with RPMI 8226 cells; after tumors appeared, the animals were treated with either the EGFR inhibitor erlotinib or vehicle until day 40. In vehicle-treated animals, tumors grew exponentially, while in erlotinib-treated animals there was little change in tumor volume; this difference was significant on day 40 (Figure 6A,B). Estimated weights of excised tumors were significantly lower in erlotinib-treated animals (Figure 6C). 

Immunohistochemical analyses of tumor slices revealed that erlotinib treatment significantly reduced the number of endothelial cells staining positively for CD31, a marker of microvessel density (Figure 6D,E). Erlotinib treatment also reduced the percentage of cells staining positively for Ki-67, a marker of proliferation (Figure 6F,G). Altogether these observations clearly show that erlotinib reduces tumor growth, tumor microvessel density, and tumor cell proliferation. 

### 2.7. Clinical Correlates and Prognostic Significance (CoMMpass Study)

**Figure 7:**
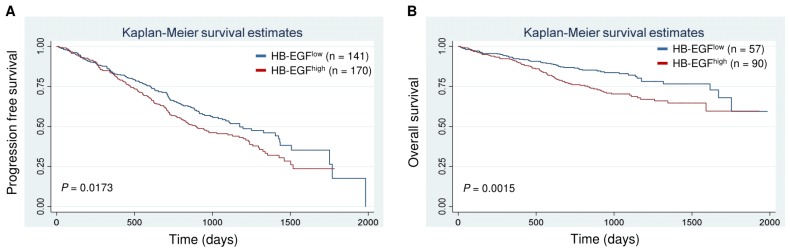
HB-EGF expression predicts survival in MM patients. Kaplan–Meier survival curves for cases in the lowest vs. highest quintiles of HB-EGF expression among 647 patients of the MMRF cohort (https://research.themmrf.org). Patients with higher HB-EGF expression have shorter progression-free survival (**A**) and overall survival (**B**) Log-rank test.

Finally, to assess the clinical implications of HB-EGF overexpression, we analyzed the association between HB-EGF expression levels at MM diagnosis and survival in patients enrolled in the CoMMpass study [24]. For this purpose, we compared patients in the first quintile of HB-EGF expression (i.e., the 20% with the lowest levels) with those of the fifth quintile (i.e., the 20% with the highest levels). Kaplan–Meier survival analyses revealed shorter progression-free survival (*p* = 0.0173) and overall survival (*p* = 0.0015) in patients in the fifth quintile (Figure 7A,B).

## 3. Discussion

This study revealed that EGFR is expressed on bone marrow endothelial cells from MGUS patients (MGEC) and MM patients (MMEC), although at higher levels on the latter. The study also identified HB-EGF as the main autocrine EGFR ligand produced by MMEC. This evidence suggests that cellular components of MM bone marrow, including endothelial cells, release HB-EGF to sustain disease progression. Moreover, we found that MMEC respond to increasing concentrations of HB-EGF in the surrounding microenvironment by augmenting EGFR protein expression. This autocrine loop is amplified by tumor plasma cells that stimulate expression of HB-EGF and EGFR in MMEC. These findings suggest that during the transition from the avascular to the vascular phase, proliferation of tumor cells and changes in the bone marrow microenvironment stimulate the upregulation of both HB-EGF and EGFR in MMEC.

Our study also documented that HB-EGF–EGFR signaling is involved in MM-associated angiogenesis, and that this process is restrained by EGFR inhibition in vitro and in vivo. In particular, in MMEC, the EGFR inhibitor erlotinib reduced new blood vessel formation and prevented tumor plasma cell growth in vitro and in vivo. These findings encourage the testing of this drug in combination therapies for MM, in line with existing treatment regimens for several solid tumors [25,26,27]. 

There are some caveats associated with the current study. First, HB-EGF is also produced by tumor plasma cells. This may have biased our results given that it is unclear how much is the contribution of these cells in the amplification of the HB-EGF–EGFR signaling. Nevertheless, data from our and other groups demonstrate that tumor plasma cells produce relatively low quantities of HB-EGF that are negligible compared with those produced by bone marrow stromal cells such as endothelial cells [16]. Second, not only RPMI-8226 cells but also U266 cells express EGFR and produce HB-EGF. However, only RPMI-8226 cells harbor KRAS mutations which have been shown to predict patient response to EGFR inhibitors in solid tumors [28].

To our knowledge, this is the first report that clearly links HB-EGF–EGFR signaling to the angiogenic switch of the MGUS-to-MM transition. So far, enhancement of HB-EGF expression in carcinogenesis has only been found in solid tumors under hypoxic conditions [29,30,31,32]. As shown in ameloblastoma, transcription and post-transcription factors such as hypoxia-inducible factor 1α (HIF-1α), ADAM-12, and NOTCH1 are directly involved in an HB-EGF autocrine amplification loop and facilitate local tumor invasiveness [29].

A recent report demonstrated that EGFR signaling influences proteasome activity by modulating the expression of the immunoproteasome subunits LMP7 and LMP2 in tumor plasma cells. LMP7 and LMP2 levels affect proteasome capacity and sensitivity to the proteasome inhibitor bortezomib. This means that EGFR signaling impacts response to therapy and clinical outcome in MM patients. Modulation of LMP7 and LMP2 expression occurs through tight junction protein 1 (TJP1), a regulator of the EGFR/JAK1/STAT3 pathway. TJP1 binds to EGFR and inhibits EGFR/JAK1/STAT3 signaling with subsequent downregulation of LMP7 and LMP2 expression [33]. In this perspective, erlotinib could readily be moved to MM treatment, especially in patients whose plasma cells have high surface EGFR expression.

It is also interesting to note that the mutational state of EGFR and the activation of the EGFR/Ras/ERK pathway associates with the central nervous system (CNS) tropism of several tumors [34,35,36,37] and that hyperstimulation of several tyrosine kinases and mitogen-activated protein kinases have been found in MM patients with aggressive disease and CNS involvement [38,39]. These patients that are usually p53 and/or BRAF mutated [40,41] show clinical response to pomalidomide [42] and could benefit from a combination of pomalidomide (or other IMiDs) and EGFR-targeted agents, such as cetuximab [43]. Ongoing and planned studies will further address this possibility as we fully explore the biology of the EGFR pathway in MM.

## 4. Materials and Methods

### 4.1. Biological Materials

Heparinized bone marrow aspirates and iliac crest biopsies were obtained from patients fulfilling the International Myeloma Working Group criteria [44] for MM and MGUS. The MM patients (22 men and 10 women; aged from 50 to 80 years; median, 65 years) were at first diagnosis. The MGUS patients (14 men and 6 women; aged from 48 to 78 years; median, 63 years) had IgG MGUS (*n* = 13), IgA MGUS (*n* = 5), and k or λ MGUS (*n* = 2). The study protocol was approved by the Ethical Committee of the University of Bari Medical School, and all patients provided their written informed consent in accordance with the Declaration of Helsinki. 

### 4.2. Cell Isolation and Culture Procedures

Heparinized bone marrow aspirates from MM and MGUS patients were used to isolate bone marrow mononuclear cells (BMMC) and, from these, primary endothelial cells (MMEC and MGEC). Cells were separated and cultured as described previously [45] and detailed in Supplementary Methods. The purity of cell populations was determined using the FACSCanto II flow cytometry system (Becton Dickinson-BD, San Jose, CA, USA) and found to be >95% in all cases. Percentages of plasma cells in BMMC from MGUS and MM patients were 0.05–0.10% and 10–20%, respectively. 

Human RPMI 8266 MM cells were obtained from the American Type Culture Collection (Rockville, MD, USA) and cultured in RPMI-1640 medium supplemented with 10% fetal bovine serum (FBS). The cells routinely tested negative for mycoplasma contamination.

In coculture experiments, MMEC (4 × 10^5^ cells/100 mm Petri dish) were cultured alone or with RPMI 8226 cells at 1:1 and 1:10 cell ratios, together or separated by a Transwell membrane (0.4 μm pore size; Corning, New York, NY, USA). After 24 h, aliquots of supernatant were collected and frozen at −80°C for ELISA, and MMECs were harvested for western blotting and quantitative real-time PCR. For cocultures without the Transwell membrane, MMEC were immunomagnetically purified using CD31 Microbeads (Miltenyi Biotec, Bergisch Gladbach, Germany) to eliminate RPMI 8226 cells.

### 4.3. Conditioned Media

Supernatants of 7-day cultured BMMC were collected and used as conditioned culture medium (MGUS BM medium or MM BM medium) for treating adherent MMEC for 24 h; they were also used in ELISAs for HB-EGF determination. 

Supernatants of 24 h serum-free cultured MMEC, treated or not with 100 ng/mL recombinant HB-EGF, served as MMEC-conditioned medium in membrane-based sandwich immunoassays (Human Angiogenesis Array kits; R&D Systems) and in chorioallantoic membrane assays, as detailed in Supplementary Methods. 

### 4.4. Real-Time PCR

Total RNA from MMEC and MGEC was extracted using the RNeasy Mini kit (Qiagen), and used to prepare cDNA with the iScript cDNA Synthesis Kit (Bio-Rad). Levels of mRNA for genes encoding EGFR, HER4, HB-EGF (*HBEGF*), EGF, TGF-α, betacellulin (*BTC* gene), epiregulin (*EREG*), amphiregulin (*AREG*), MMP3, MMP7, ADAM10, ADAM12, ADAM17 and glyceraldehyde 3-phosphate dehydrogenase (*GAPDH*) were determined in triplicate by real-time PCR using TaqMan probes (Applied Biosystems assay IDs are reported in Table S1). Amplification and fluorescence reading were performed on a StepOne real-time PCR system (Applied Biosystems). Relative quantification of mRNA levels was done using the comparative Ct method with *GAPDH* as reference gene and with the 2^−ΔΔCT^ formula.

### 4.5. Immunochemical Procedures

Western blotting, immunofluorescence, and immunohistochemistry procedures were done using the antibodies listed in Table S2 according to methods described in the Supplementary Methods. 

### 4.6. HB-EGF Determination via ELISA

Aliquots of culture medium from MMEC, BMMC, and MMEC–RPMI 8226 cocultures were analyzed for soluble HB-EGF using Human HB-EGF ELISA kits (Thermo Fisher Scientific; cat. no. EHHBEGF). The assays were read using a Bio-Rad 3550 plate reader.

### 4.7. Functional Assays

For functional assays, recombinant human HB-EGF, soluble form (PeproTech, Rocky Hill, NJ, USA), was dissolved at 1 mg/mL in sterile water. The neutralizing-blocking polyclonal goat anti-HB-EGF IgG (cat. no. AF-259-NA) was purchased from R&D Systems (Minneapolis, MN, USA). The EGFR inhibitor erlotinib (Selleck Chemicals, Houston, TX, USA) was dissolved at 75 mg/mL in DMSO (Sigma-Aldrich). Matrigel Basement Membrane Matrix was purchased from Becton Dickinson-BD Biosciences (San Jose, CA, USA). These reagents were further diluted in serum-free culture medium as needed.

Wound-healing assays were performed as previously described [46] and detailed in Supplementary Methods. For in vitro angiogenesis assays, MMEC were plated on Matrigel-coated 48-well plates in serum-free medium, alone or supplemented with increasing concentrations of recombinant human HB-EGF (1, 10, 100 ng/mL) for 3 h. In some experiments, MMEC were plated in serum-free medium containing 0.5 μg/mL anti-HB-EGF antibody or 10 μM erlotinib for 12 h. Photomicrographs of skeletonized meshes were acquired in three randomly chosen fields with an EVOS microscope. Angiogenic behavior was determined by measuring the topological parameters vessel length, mesh area, and number of branching points using a computerized image analyzer as described by Guidolin et al. [47]. Data were normalized to control values.

The chorioallantoic membrane assay was performed as previously described [48] and detailed in Supplementary Methods.

### 4.8. RNA Interference

MMECs (4 × 10^5^ cells/100 mm Petri dish) were transiently transfected with 50 nM EGFR siRNA (small interfering RNA) or control non-targeting siRNA (SMARTpool; Thermo Fisher) using the transfection reagent Lipofectamine (Life Technologies) for 48 h. siRNA-transfected MMEC were evaluated immediately in western blot and angiogenesis assays.

### 4.9. MM Xenograft Model

Twenty female, 6- to 8-week-old non-obese diabetic/severe combined immunodeficiency mice (NOD.CB17-Prkdcscid/NCrHsd; Envigo, Huntingdon, UK) were each injected subcutaneously, in the right flank, with 10^7^ RPMI 8226 cells suspended in 400 μL of a 50:50 Matrigel–RPMI-1640 medium solution. Three days later, tumor-bearing mice were randomized to once-daily intraperitoneal erlotinib (50 mg/kg, 5 days per week) or vehicle (10% DMSO, 10% Tween 80 in sterile water; control group), and treated until day 39. Tumor length and width (in mm) were measured every 3 days, and weight (in mg) was estimated from the formula (length × width^2^)/2 (assuming 1 mg = 1 mm^3^). Mice were killed on day 40 (when the estimated tumor weight in some animals reached the ethical limit of 2.5 g). Tumor tissue samples were fixed in formalin, embedded in paraffin, cut in 5 μm sections and processed for immunohistochemical analysis of microvessel density and cell proliferation using enzyme labeled-antibodies against murine CD31 and Ki-67 (Table S2). To quantify positively stained cells, each tumor was evaluated on five different slides and each slide was evaluated in five fields; positive cells were expressed as a percentage of all cells.

Mice were housed, treated, and killed according to the protocol approved by the Institutional Animal Care and Use Committee of the University Medical School of Bari (license n. 578/2016PR). Animals were monitored daily for clinical signs of toxicity, while body weight was measured twice weekly. The dose of erlotinib used here, 50 mg/kg, is known to be well tolerated in mice [49].

### 4.10. Survival Analysis of CoMMpass Data

Clinical and molecular data from the CoMMpass (Clinical Outcomes in Multiple Myeloma to Personal Assessment of Genetic Profiles) Study [24] were obtained from the Multiple Myeloma Research Foundation (https://research.themmrf.org). Data included overall and progression-free survival and HB-EGF RNA sequencing (RNA-seq) data from CD138^+^ cells isolated from bone marrow aspirates of 647 newly diagnosed MM patients. Normalized expression levels of HB-EGF were determined using the FPKM (fragments per kilobase of exon model per million reads mapped) method [50], and then used to divide patients into quintiles. We then compared survival data between the first quintile (i.e., the 20% of patients with the lowest HB-EGF expression) and the fifth quintile (i.e., the 20% with the highest HB-EGF expression). Survival curves were calculated using the Kaplan–Meier method and compared using the log-rank test.

### 4.11. Statistical Analyses

Nonparametric statistics were used. Tests included the Mann–Whitney U test and Wilcoxon signed-rank test. Statistical analysis was performed using Prism 5 software (GraphPad). A *p* < 0.05 was considered statistically significant.

For in vivo experiments, sample size was calculated using G*Power software version 3.1.9.2. Assuming an effect size of 0.4 with an alpha level of significance of 0.05 and a power of 80%, we determined that 9 mice were needed for each group, for a total of 18 mice. This number was increased to 20 considering an expected failure rate of 10% for each group.

## Supplementary Materials

The following are available online at https://www.mdpi.com/2072-6694/12/1/173/s1, Figure S1: Relative mRNA levels of MMP7 in MMEC after indirect and direct coculture with RPMI 8226 cells, Figure S2: Supernatant of RPMI 8226 cocultures stimulates in vitro MMEC migration and angiogenesis, Figure S3: HB-EGF modulates the secretion of angiogenesis-related proteins by MMEC, Figure S4: MM plasma cells do not affect HER4 expression on bone marrow endothelial cells, Table S1: Taqman qRT-PCR probes used for gene expression analyses, Table S2: Antibodies against human and murine antigens used in immunochemical procedures.
