# Supplementary material for: HB-EGF–EGFR Signaling in Bone Marrow Endothelial Cells Mediates Angiogenesis Associated with Multiple Myeloma
Source: Cancers (Basel). 2020 Jan 10;12(1):173. doi: 10.3390/cancers12010173 (PMC7017291; doi:10.3390/cancers12010173)
Supplement: Supplementary file 1 [file cancers-12-00173-s001.zip › Supplementary Materials.pdf]

cancers MDPI

Supplementary Materials

HB-EGF–EGFR Signaling in Bone Marrow Endothelial Cells Mediates Angiogenesis Associated with Multiple Myeloma

Luigia Rao, Donato Giannico, Patrizia Leone, Antonio Giovanni Solimando, Eugenio Maiorano, Concetta Caporusso, Loren Duda, Roberto Tamma, Rosanna Mallamaci, Nicola Susca, Alessio Buonavoglia, Matteo Claudio Da Vià, Domenico Ribatti, Vallì De Re, Angelo Vacca and Vito Racanelli

Supplementary Methods

Cell Separation and Culture Procedures

Bone marrow mononuclear cells (BMMC) were obtained by centrifugation of heparinized bone marrow aspirates on Ficoll-Paque Plus gradients (GE Healthcare Bio-Science AB, Uppsala, Sweden) and maintained in Roswell Park Memorial Institute (RPMI)-1640 medium supplemented with 10% heat inactivated fetal bovine serum (FBS).

MGEC and MMEC were isolated from BMMC using CD31 Microbeads (Miltenyi Biotec) and grown in Dulbecco's modified Eagle's medium (DMEM) supplemented with 20% heat-inactivated FBS, 2 mM L-glutamine, 100 U/mL penicillin, and 100 µg/mL streptomycin (all from Sigma-Aldrich; culture medium). In functional studies, MMEC were used until the sixth passage of culture.

MMEC were also grown in serum-free DMEM for 24 hours to obtain supernatants to be used as conditioned media (MMEC conditioned medium) in CAM assays and angiogenesis arrays.

Trypsin-EDTA and PBS without Ca^{2+} and Mg^{2+} were purchased from Sigma-Aldrich.

Western Blotting

MGEC and MMEC cells were lysed by using RIPA Lysis and Extraction Buffer (Thermo Fisher Scientific). Total protein lysates (30 µg/lane) were separated on X% polyacrylamide gels under denaturing and reducing conditions, and then transferred to membranes for immunoblotting, using the antibodies indicated in Table S2.

Bound primary antibodies were detected with horseradish peroxidase–conjugated anti-mouse or anti-rabbit IgG (Bio-Rad). Immunoreactive bands were visualized by enhanced chemiluminescence using SuperSignal West Femto Maximum Sensitivity Substrate (Thermo Fisher Scientific) with a Gel Logic 1500 Imaging System (Eastman Kodak) and quantified as optical density units with Kodak Molecular Imaging Software. Results were expressed as relative density.

Immunofluorescence

MGEC or MMEC (5×10^3) were cultured on chamber slides (Lab-Tek) for 48 h. Cells were washed with PBS, fixed with 2% paraformaldehyde, permeabilized with 0.2% Triton X-100 (Sigma-Aldrich), and incubated with anti-EGFR antibody (Cell Signaling Technology; cat. no. 4267), and then with goat anti-rabbit IgG-TRITC (Sigma-Aldrich). Nuclei were counterstained with 4′,6-diamidino-2-phenylindole (DAPI, Thermo Fisher Scientific). Slides were examined under a fluorescence microscope (Olympus).

Immunohistochemistry

Iliac crest biopsies from MM and MGUS patients were fixed, decalcified, processed into 4-µm sections, and stained with mouse anti-human EGFR mAb (DAKO-Agilent; cat. no. M7239) and anti-CD31 pAb (Abcam; cat. no. ab32457) using a biotin-streptavidin method. Microphotographs were acquired in three or four fields (at 400X magnification) spanning the entirety of three sections per sample.

"Wound" healing" Assay

MMEC were grown until confluence on fibronectin-coated (10 mg/mL) 12 well plate and the "wound" was made by scraping the cell monolayer with a P200 pipette tip. Cells were exposed to serum free medium (SFM) alone or admixed with increasing concentrations of human HB-EGF (1, 10, 100 ng/mL) (PEPROTECH). MMEC were also treated with 0.5 µg/mL neutralizing/blocking anti-HB-EGF Ab (R&D system, cat. no. AF-259-NA) or with 10 µM Erlotinib (Selleck Chemicals). Afterward cells were fixed with 4% paraformaldehyde and stained with crystal violet (all from Sigma-Aldrich). The migrating MMEC were counted into 3 different fields of the wound area of each 10× field with EVOS digital inverted microscope (Euroclone, Pero, MI, Italy).

Human Angiogenesis Array

MMEC were cultured in SFM with or without 100 ng/mL HB-EGF for 24 h and media were collected and concentrated to be analyzed by Human Angiogenesis Array kit (R&D System) according to the manufacturer's instructions. Spots were quantified with Image Lab 5.1 Software (Bio-Rad) and values were reported as mean pixel density.

Chorioallantoic Membrane Assay (CAM)

Fertilized white Leghorn chicken eggs were incubated at 37 °C at constant humidity. On day 3, the shell was opened and 2 to 3 mL of albumen was removed to detach the chorioallantoic membrane (CAM). On the 8th day, the CAM were implanted with 1 mm^3 sterilized gelatin sponges (Gelfoam, Upjohn Co, MI, USA) filled with SFM alone or with 0.5 µg/mL of neutralizing/blocking anti-HB-EGF Ab (cat. no. AF-259-NA), or with MMEC CM in presence or absence of 0.5 µg/mL of neutralizing/blocking anti-HB-EGF Ab, or with medium of HB-EGF-treated MMEC (HB-EGF CM) with or without neutralizing/blocking anti-HB-EGF. On the 12th day, blood vessels entering the sponges within the focal plane of the CAM were counted and pictures were taken in *ovo* at 50× (Olympus stereomicroscope).

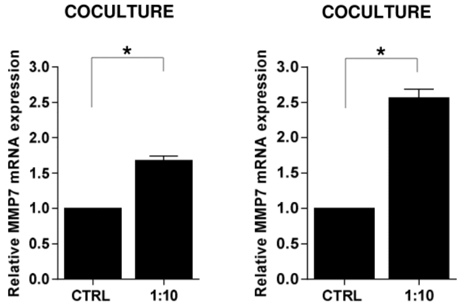

Figure S1. Relative mRNA levels of MMP7 in MMEC after indirect and direct coculture with RPMI 8226 cells. Samples from six patients were tested in triplicate.

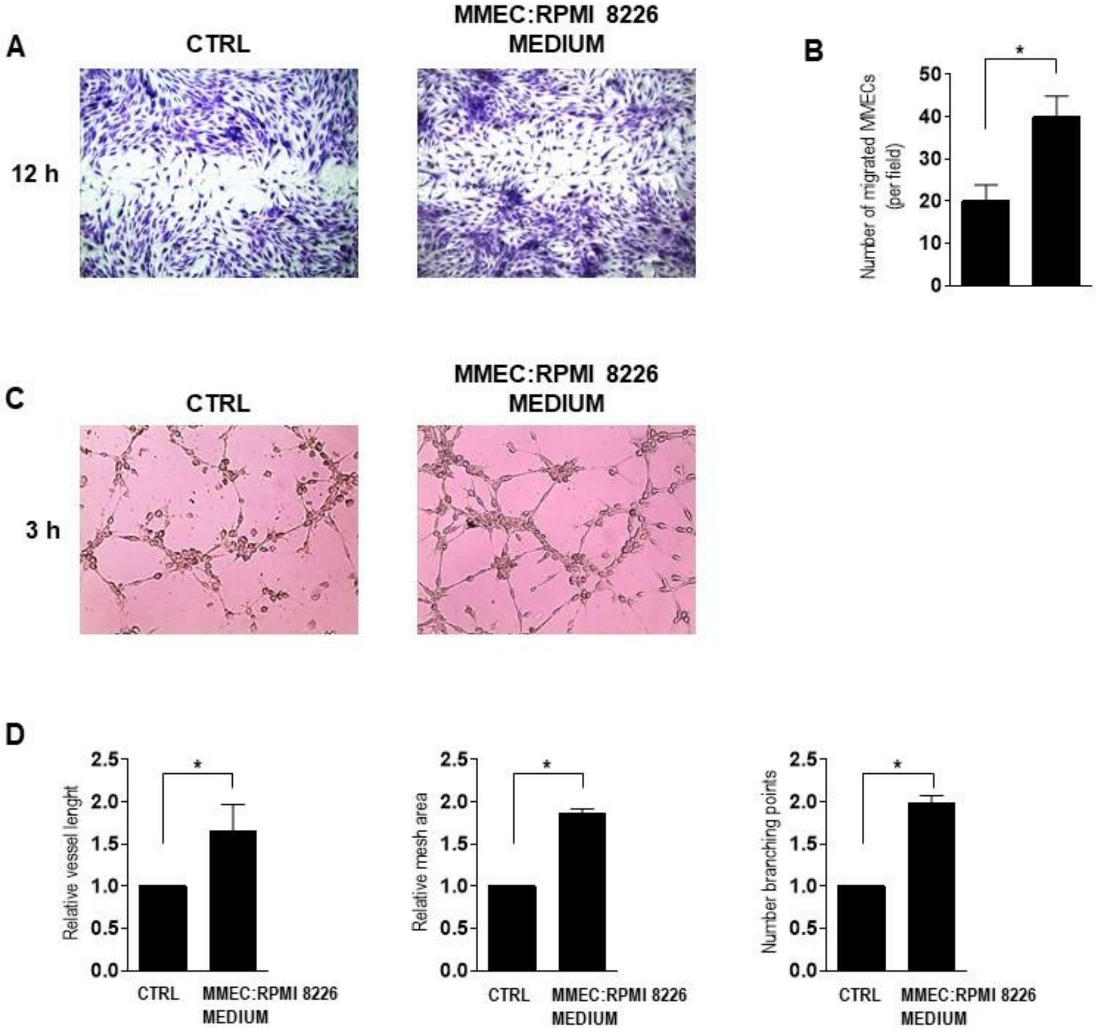

Figure S2. Supernatant of RPMI 8226 cocultures stimulates in vitro MMEC migration and angiogenesis. (**A**,**B**) Wound-healing assay. (**A**) Photomicrographs of MMEC treated with supernatant of RPMI 8226 cocultures, 12 h after confluent monolayers were wounded by scraping. (**B**) Counts of migrating cells in each wound of (A), for six independent experiments. (**C**,**D**) Matrigel angiogenesis assay. (**C**) Photomicrographs of MMEC, 3 h after seeding on Matrigel in serum-free medium supplemented with supernatant of RPMI 8226 cocultures. Images are representative of experiments using cells from six patients. (**D**) Quantification of angiogenic behavior in (C) by topological analysis. Original magnification, 200×. Scale bar, 50 µm. Data are mean and SD of six independent experiments. * $p < 0.05$, Mann-Whitney U test.

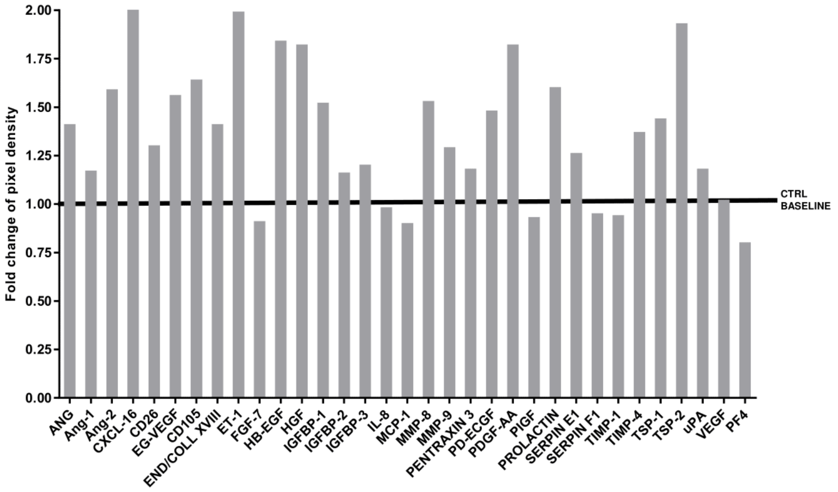

Figure S3. HB-EGF modulates the secretion of angiogenesis-related proteins by MMEC. 24-h serum-free media conditioned by MMEC, in the absence or presence of 100 ng/ml recombinant HB-EGF, were analyzed for the presence of 55 angiogenesis-related proteins by membrane-based sandwich immunoassay. Relative levels of proteins in HB-EGF-treated samples were expressed as fold change relative to the average value for untreated samples. Only proteins exceeding the detection limit of the assay are shown.

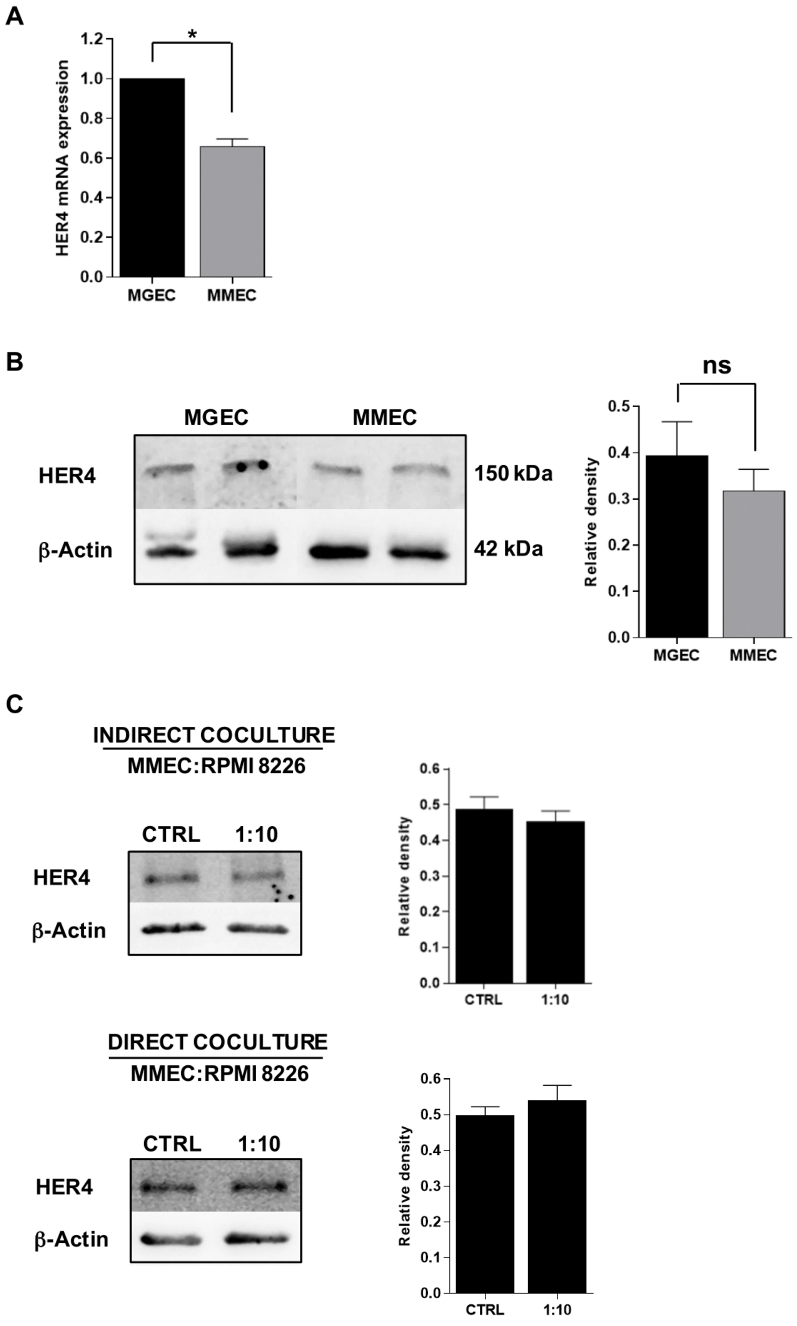

Figure S4. MM plasma cells do not affect HER4 expression on bone marrow endothelial cells. (**A**) Relative mRNA expression levels of HER4 in MGEC and MMEC. Values were normalized to MGEC. (**B**) Western blots of HER4 and β-actin in MGEC and MMEC lysates (left) and densitometric analysis of HER4 normalized to β-actin. (**C**) Western blots of MMEC cocultured indirectly or directly with RPMI 8226 cells or cultured alone for 24 h and densitometric quantification of HER4 normalized to β-actin. Samples from six MGUS and six MM patients were tested in triplicate. Data are expressed as mean and SD. * $p < 0.05$, Mann-Whitney U test.

Table S1. Taqman qRT-PCR probes used for gene expression analyses.

Gene Symbol	Probe ID
EGFR (epidermal growth factor receptor)	Hs01076090_m1
HER4 (Receptor tyrosine-protein kinase erbB-4)	Hs00955522_m1
HB-EGF (Heparin-binding EGF-like growth factor)	Hs00181813_m1
EGF (Epidermal growth factor)	Hs01099990_m1
TGF-α (Transforming growth factor alpha)	Hs00608187_m1
BTC (Betacellulin)	Hs01101201_m1
EREG (Epiregulin)	Hs00914313_m1
AREG (Amphiregulin)	Hs00950669_m1
MMP3 (matrix metallopeptidase 3)	Hs00968305_m1
MMP7 (matrix metallopeptidase 7)	Hs01042796_m1
ADAM10 (ADAM metallopeptidase domain 10)	Hs00153853_m1
ADAM12 (ADAM metallopeptidase domain 12)	Hs01106101_m1
ADAM17 (ADAM metallopeptidase domain 17)	Hs01041915_m1
GAPDH (Glyceraldehyde 3-phosphate dehydrogenase)	Hs02786624_g1

Table S2. Antibodies against human and murine antigens used in immunochemical procedures.

Antigen (Species)	Host Species	Vendor (Catalog Number)	Dilution
CD31 (human)	Rabbit	Abcam (ab32457)	1:2000
CD31 (mouse)	Rabbit	Abcam (ab124432)	1:1000
EGFR (human)	Rabbit	Cell Signaling Technology (4267)	1:1000
EGFR (human)	Mouse	Dako-Agilent (M7239)	1:50
HB-EGF (human)	Goat	R&D Systems (AF-259-NA)	1:2000
HB-EGF (human)	Mouse	Abcam (ab66792)	1:1000
HER4 (human)	Rabbit	Cell Signaling Technology (4795)	1:1000
Ki-67 (human)	Mouse	Dako-Agilent (M7240)	1:100
beta-Actin (human)	Mouse	Sigma-Aldrich (A1978)	1:10000

© 2020 by the authors. Licensee MDPI, Basel, Switzerland. This article is an open access article distributed under the terms and conditions of the Creative Commons Attribution (CC BY) license (http://creativecommons.org/licenses/by/4.0/).
