# Supplementary material for: HB-EGF–EGFR Signaling in Bone Marrow Endothelial Cells Mediates Angiogenesis Associated with Multiple Myeloma
Source: Cancers (Basel). 2020 Jan 10;12(1):173. doi: 10.3390/cancers12010173 (PMC7017291; doi:10.3390/cancers12010173)
Supplement: Supplementary file 1 [file cancers-12-00173-s001.zip › WB Figures.pdf]

FIG. 1B

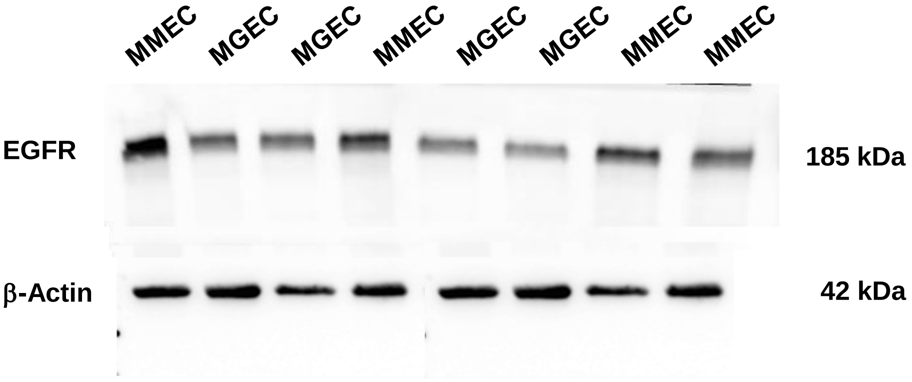

FIG. 2A

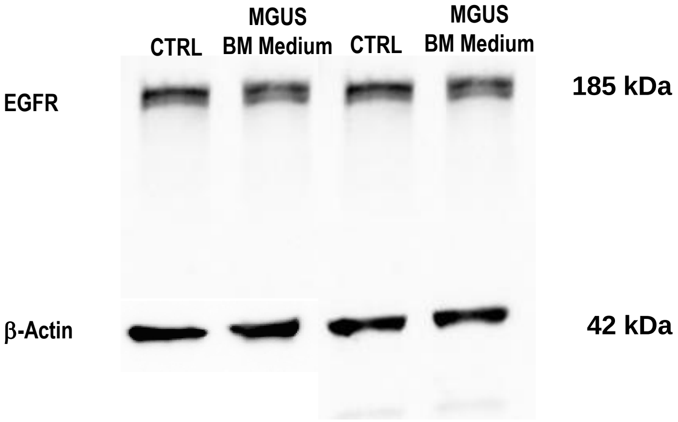

FIG. 2B

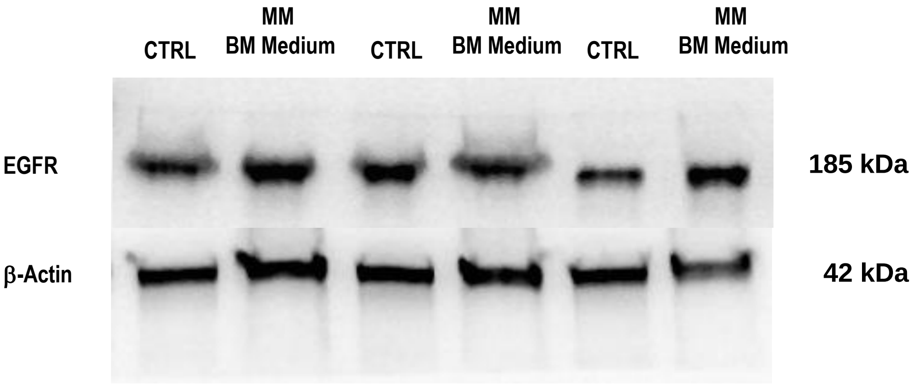

FIG. 2C

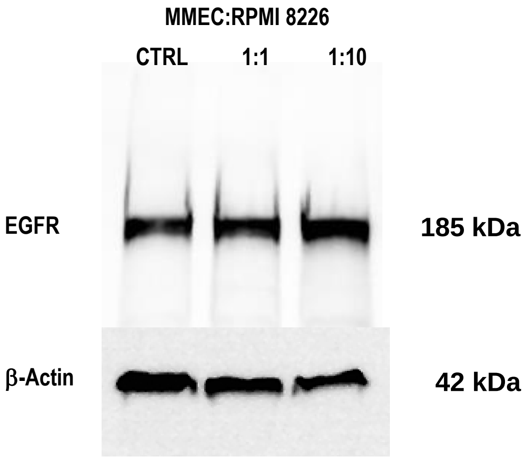

FIG. 2D

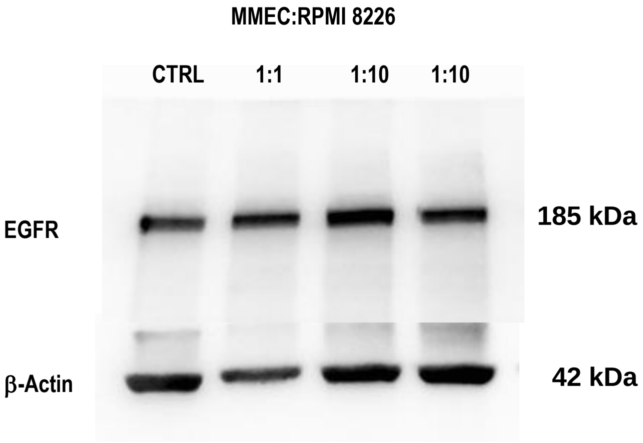

FIG. 2E

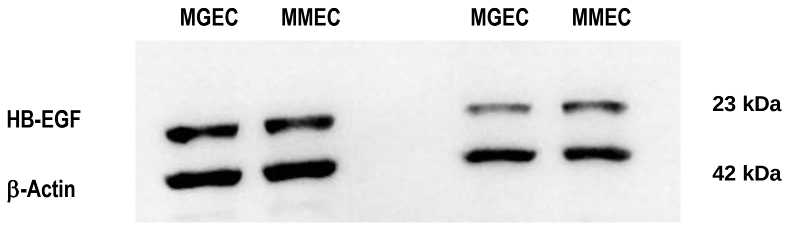

FIG. 5E

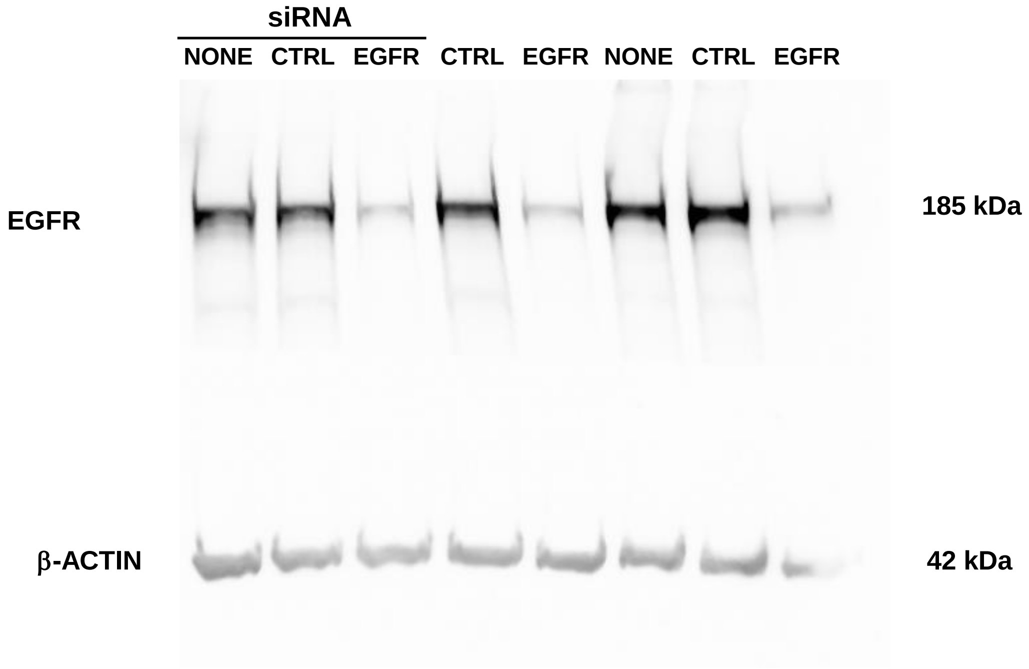

In each western blotting analysis we usually insert pre-stained standard markers with a defined molecular weight. Since these markers are not chemiluminescent and are not visible during detection, we use to make small signs in correspondence to the markers before the immunodetection. Therefore, we have included the molecular weights in the western blots of supplemental materials.
